# Diaqua­(isonicotinamide-κ*N* ^1^)(4-meth­oxy­benzoato-κ^2^ *O*,*O*′)(4-meth­oxy­benzoato-κ*O*)cobalt(II)

**DOI:** 10.1107/S160053681002194X

**Published:** 2010-06-16

**Authors:** Tuncer Hökelek, Yasemin Süzen, Barış Tercan, Erdinç Tenlik, Hacali Necefoğlu

**Affiliations:** a Department of Physics, Hacettepe University, 06800 Beytepe, Ankara, Turkey; b Department of Chemistry, Faculty of Science, Anadolu University, 26470 Yenibağlar, Eskişehir, Turkey; c Department of Physics, Karabük University, 78050 Karabük, Turkey; d Department of Chemistry, Kafkas University, 63100 Kars, Turkey

## Abstract

In the title complex, [Co(C_8_H_7_O_3_)_2_(C_6_H_6_N_2_O)(H_2_O)_2_], the Co^II^ atom is coordinated by three O atoms from two 4-meth­oxy­benzoate ligands, which act in different modes, *viz.* monodentate and bidentate, two water mol­ecules and one N atom of the isonicotinamide ligand in a distorted octa­hedral geometry. The monodentate-coordinated carboxyl­ate group is involved in an intra­molecular O—H⋯O hydrogen bond with the coordinated water mol­ecule. In the crystal structure, inter­molecular O—H⋯O and N—H⋯O hydrogen bonds link the mol­ecules into layers parallel to the *ab* plane. The crystal packing is further stabilized by weak C—H⋯O hydrogen bonds and π–π inter­actions indicated by the short distance of 3.6181 (8) Å between the centroids of the benzene and pyridine rings of neighbouring mol­ecules.

## Related literature

For general background to niacin and the nicotinic acid deriv­ative *N*,*N*-diethyl­nicotinamide, see: Krishnamachari (1974 ▶) and Bigoli *et al.* (1972 ▶), respectively. For related structures, see: Greenaway *et al.* (1984 ▶); Hökelek *et al.* (2009*a* ▶,*b* ▶,*c* ▶,*d* ▶); Necefoğlu *et al.* (2010 ▶).
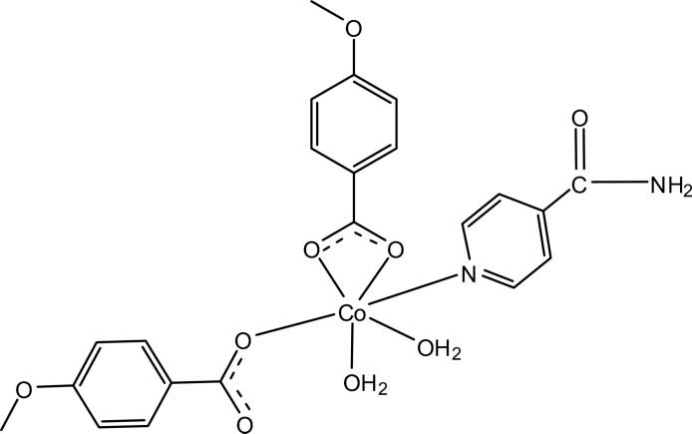


## Experimental

### 

#### Crystal data

[Co(C_8_H_7_O_3_)_2_(C_6_H_6_N_2_O)(H_2_O)_2_]
*M* *_r_* = 519.36
Monoclinic, 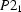

*a* = 8.2666 (2) Å
*b* = 6.8055 (2) Å
*c* = 20.5415 (4) Å
β = 99.808 (2)°
*V* = 1138.74 (5) Å^3^
*Z* = 2
Mo *K*α radiation
μ = 0.81 mm^−1^
*T* = 100 K
0.39 × 0.32 × 0.28 mm

#### Data collection

Bruker Kappa APEXII CCD area-detector diffractometer
Absorption correction: multi-scan (*SADABS*; Bruker, 2005 ▶) *T*_min_ = 0.739, *T*_max_ = 0.791
11263 measured reflections
4838 independent reflections
4597 reflections with *I* > 2σ(*I*)
*R*_int_ = 0.020

#### Refinement

*R*[*F* ^2^ > 2σ(*F* ^2^)] = 0.022
*wR*(*F* ^2^) = 0.050
*S* = 1.01
4838 reflections
333 parameters
H atoms treated by a mixture of independent and constrained refinement
Δρ_max_ = 0.29 e Å^−3^
Δρ_min_ = −0.24 e Å^−3^
Absolute structure: Flack (1983 ▶), 1761 Friedel pairs
Flack parameter: 0.015 (7)

### 

Data collection: *APEX2* (Bruker, 2007 ▶); cell refinement: *SAINT* (Bruker, 2007 ▶); data reduction: *SAINT*; program(s) used to solve structure: *SHELXS97* (Sheldrick, 2008 ▶); program(s) used to refine structure: *SHELXL97* (Sheldrick, 2008 ▶); molecular graphics: *ORTEP-3 for Windows* (Farrugia, 1997 ▶); software used to prepare material for publication: *WinGX* (Farrugia, 1999 ▶) and *PLATON* (Spek, 2009 ▶).

## Supplementary Material

Crystal structure: contains datablocks I, global. DOI: 10.1107/S160053681002194X/cv2727sup1.cif

Structure factors: contains datablocks I. DOI: 10.1107/S160053681002194X/cv2727Isup2.hkl

Additional supplementary materials:  crystallographic information; 3D view; checkCIF report

## Figures and Tables

**Table 1:** Hydrogen-bond geometry (Å, °)

*D*—H⋯*A*	*D*—H	H⋯*A*	*D*⋯*A*	*D*—H⋯*A*
N2—H2*A*⋯O2^i^	0.79 (3)	2.11 (3)	2.877 (2)	164.0 (17)
N2—H2*B*⋯O1^ii^	0.91 (3)	2.16 (3)	3.050 (2)	167 (2)
O8—H81⋯O4	0.83 (3)	1.84 (3)	2.6577 (17)	167 (3)
O8—H82⋯O7^iii^	0.89 (2)	1.86 (3)	2.7427 (16)	172 (2)
O9—H91⋯O6^iv^	0.786 (19)	2.078 (19)	2.8384 (16)	163 (2)
O9—H92⋯O4^v^	0.91 (3)	1.72 (3)	2.6307 (18)	174.1 (15)
C8—H8*A*⋯O7^vi^	0.96	2.53	3.466 (2)	166
C16—H16*B*⋯O4^vii^	0.96	2.52	3.4752 (18)	171

Symmetry codes: (i) 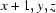; (ii) 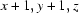; (iii) 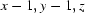; (iv) 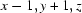; (v) 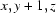; (vi) 
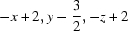
; (vii) 
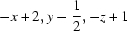
.

**Fig. 1.**
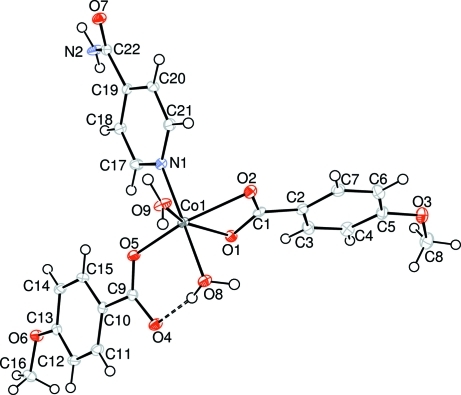
The molecular structure of (I) with the atom-numbering scheme. Displacement ellipsoids are drawn at the 50% probability level. Dashed line indicates the hydrogen-bonding.

## supplementary crystallographic information

### Comment

As a part of our ongoing investigation on transition metal complexes of nicotinamide (NA), one form of niacin (Krishnamachari, 1974), and/or the nicotinic acid derivative *N*,*N*-diethylnicotinamide (DENA), an important respiratory stimulant (Bigoli *et al.*, 1972), the title compound has been synthesized. Herein we report its crystal structure.

The title compound, (I), is a monomeric complex, where the Co^II^ ion is surrounded by two methoxybenzoate (MB) anions, one isonicotinamide (INA) ligand and two coordinated water molecules. One of the MB anions acts as a bidentate ligand, while the other is monodentate. The structures of similar complexes , [Mn(C_9_H_10_NO_2_)_2_(C_6_H_6_N_2_O)(H_2_O)_2_] (II) (Hökelek *et al.*, 2009*a*), [Co(C_9_H_10_NO_2_)_2_(C_6_H_6_N_2_O)(H_2_O)_2_] (III) (Hökelek *et al.*, 2009*b*), [Cd(C_8_H_7_O_2_)_2_(C_6_H_6_N_2_O)_2_(H_2_O)].H_2_O (IV) (Necefoğlu *et al.*, 2010), [Zn(C_9_H_10_NO_2_)_2_(C_6_H_6_N_2_O)(H_2_O)_2_] (V) (Hökelek *et al.*, 2009*c*) and [Zn(C_8_H_8_NO_2_)_2_(C_6_H_6_N_2_O)_2_].H_2_O (VI) (Hökelek *et al.*, 2009d) have also been determined.

In (I) (Fig. 1), the four O atoms (O1, O2, O5 and O9) in the equatorial plane around the Co1  form a highly distorted square-planar arrangement, while the distorted octahedral coordination geometry is completed by the N atom (N1) of INA ligand and the O atom (O8) of the second water molecule in the axial positions. The average Co—O bond length is 2.1171 (12) Å and the Co atom is displaced out of the least-squares planes of the carboxylate groups (O1/C1/O2) and (O4/C9/O5) by -0.0061 (2) Å and -0.5367 (2) Å, respectively. The dihedral angle between the planar carboxylate groups and the adjacent benzene rings A (C2—C7) and B (C9—C14) are 12.12 (12)° and 9.26 (13)°, respectively, while those between rings A, B and C (N1/C17—C21) are A/B = 78.18 (4), A/C = 74.20 (5) and B/C = 6.23 (5) °. The intramolecular O—H···O hydrogen bond (Table 1) between the monodentate-coordinated carboxyl group and a coordinated water molecule results in a six-membered ring D (Co1/O4/O5/O8/C9/H81) adopting envelope conformation, with atom Co1 displaced by -0.5481 (2) Å from the plane of the other ring atoms. In (I), the O1—Co1—O2 angle is 60.32 (4)°. The corresponding O—M—O (where *M* is a metal) angles are 54.71 (4)° in (IV), 60.03 (6)° in (V), 59.02 (8)° in (VI) and 55.2 (1)° in [Cu(Asp)_2_(py)_2_] (where Asp is acetylsalicylate and py is pyridine) [(VII); Greenaway *et al.*, 1984].

In the crystal structure, intermolecular O—H···O and N—H···O hydrogen bonds (Table 1) link the molecules into layers parallel to *ab* plane. The crystal packing is further stabilized by the weak C—H···O hydrogen bonds (Table 1). The π–π contact between the benzene and pyridine rings, *Cg*2—*Cg*3^i^ [symmetry code: (i) *x*, *y* + 1, *z*, where *Cg*2 and *Cg*3 are the centroids of the rings B (C9—C14) and C (N1/C17—C21), respectively] may also stabilize the structure, with centroid-centroid distance of 3.6181 (8) Å.

### Experimental

The title compound was prepared by the reaction of CoSO_4_.7H_2_O (2.81 g, 10 mmol) in H_2_O (50 ml) and INA (2.44 g, 20 mmol) in H_2_O (50 ml) with sodium 4-methoxybenzoate (3.48 g, 20 mmol) in H_2_O (100 ml). The mixture was filtered and set aside to crystallize at ambient temperature for one week, giving brown single crystals.

### Refinement

Atoms H81, H82, H91, H92 (for water molecules) and H2A, H2B (for NH_2_) were located in difference Fourier maps and refined isotropically. The remaining H atoms were positioned geometrically with C—H = 0.93 and 0.96 Å for aromatic and methyl H atoms, respectively, and constrained to ride on their parent atoms, with *U*_iso_(H) = 1.2-1.5*U*_eq_(C).

### Crystal data

**Table:**

[Co(C_8_H_7_O_3_)_2_(C_6_H_6_N_2_O)(H_2_O)_2_]	*F*(000) = 538
*M**_r_* = 519.36	*D*_x_ = 1.515 Mg m^−^^3^
Monoclinic, *P*2_1_	Mo *K*α radiation, λ = 0.71073 Å
Hall symbol: P 2yb	Cell parameters from 6882 reflections
*a* = 8.2666 (2) Å	θ = 2.5–28.4°
*b* = 6.8055 (2) Å	µ = 0.81 mm^−^^1^
*c* = 20.5415 (4) Å	*T* = 100 K
β = 99.808 (2)°	Block, brown
*V* = 1138.74 (5) Å^3^	0.39 × 0.32 × 0.28 mm
*Z* = 2	

### Data collection

**Table:**

Bruker Kappa APEXII CCD area-detector diffractometer	4838 independent reflections
Radiation source: fine-focus sealed tube	4597 reflections with *I* > 2σ(*I*)
graphite	*R*_int_ = 0.020
φ and ω scans	θ_max_ = 28.4°, θ_min_ = 1.0°
Absorption correction: multi-scan (*SADABS*; Bruker, 2005)	*h* = −11→9
*T*_min_ = 0.739, *T*_max_ = 0.791	*k* = −8→9
11263 measured reflections	*l* = −27→27

### Refinement

**Table:**

Refinement on *F*^2^	Hydrogen site location: inferred from neighbouring sites
Least-squares matrix: full	H atoms treated by a mixture of independent and constrained refinement
*R*[*F*^2^ > 2σ(*F*^2^)] = 0.022	*w* = 1/[σ^2^(*F*_o_^2^) + (0.0215*P*)^2^] where *P* = (*F*_o_^2^ + 2*F*_c_^2^)/3
*wR*(*F*^2^) = 0.050	(Δ/σ)_max_ = 0.003
*S* = 1.01	Δρ_max_ = 0.29 e Å^−^^3^
4838 reflections	Δρ_min_ = −0.24 e Å^−^^3^
333 parameters	Absolute structure: Flack (1983), 1761 Friedel pairs
Primary atom site location: structure-invariant direct methods	Flack parameter: 0.015 (7)
Secondary atom site location: difference Fourier map	

### Special details

**Table:**

Geometry. All e.s.d.'s (except the e.s.d. in the dihedral angle between two l.s. planes) are estimated using the full covariance matrix. The cell e.s.d.'s are taken into account individually in the estimation of e.s.d.'s in distances, angles and torsion angles; correlations between e.s.d.'s in cell parameters are only used when they are defined by crystal symmetry. An approximate (isotropic) treatment of cell e.s.d.'s is used for estimating e.s.d.'s involving l.s. planes.
Refinement. Refinement of *F*^2^ against ALL reflections. The weighted *R*-factor *wR* and goodness of fit *S* are based on *F*^2^, conventional *R*-factors *R* are based on *F*, with *F* set to zero for negative *F*^2^. The threshold expression of *F*^2^ > σ(*F*^2^) is used only for calculating *R*-factors(gt) *etc*. and is not relevant to the choice of reflections for refinement. *R*-factors based on *F*^2^ are statistically about twice as large as those based on *F*, and *R*- factors based on ALL data will be even larger.

### Fractional atomic coordinates and isotropic or equivalent isotropic displacement parameters (Å^2^)

**Table:**

	*x*	*y*	*z*	*U*_iso_*/*U*_eq_	
Co1	0.83963 (2)	0.92738 (3)	0.717117 (8)	0.01106 (5)	
O1	0.87439 (13)	0.72789 (18)	0.79844 (5)	0.0132 (2)	
O2	0.75818 (13)	1.01182 (18)	0.81134 (5)	0.0145 (2)	
O3	0.68768 (15)	0.5746 (2)	1.08004 (5)	0.0212 (3)	
O4	0.78237 (13)	0.51498 (18)	0.63163 (5)	0.0156 (2)	
O5	0.95880 (13)	0.76378 (18)	0.65605 (5)	0.0146 (2)	
O6	1.43741 (13)	0.13627 (18)	0.56391 (5)	0.0160 (2)	
O7	1.44482 (14)	1.65456 (18)	0.77779 (5)	0.0160 (2)	
O8	0.60930 (14)	0.78883 (19)	0.68139 (6)	0.0147 (2)	
H81	0.652 (3)	0.692 (4)	0.6668 (10)	0.024 (6)*	
H82	0.564 (3)	0.750 (4)	0.7153 (12)	0.052 (7)*	
O9	0.72143 (15)	1.1511 (2)	0.66328 (6)	0.0177 (3)	
H91	0.639 (2)	1.126 (4)	0.6396 (10)	0.024 (6)*	
H92	0.749 (2)	1.277 (4)	0.6543 (9)	0.021 (5)*	
N1	1.05301 (16)	1.0966 (2)	0.74674 (6)	0.0127 (3)	
N2	1.62169 (17)	1.4016 (3)	0.79899 (7)	0.0175 (3)	
H2A	1.643 (2)	1.290 (4)	0.8055 (9)	0.015 (5)*	
H2B	1.708 (3)	1.486 (4)	0.8026 (10)	0.033 (6)*	
C1	0.80942 (19)	0.8473 (3)	0.83434 (7)	0.0130 (3)	
C2	0.79173 (19)	0.7843 (3)	0.90221 (7)	0.0140 (3)	
C3	0.86387 (19)	0.6109 (3)	0.92813 (7)	0.0172 (3)	
H3	0.9323	0.5420	0.9046	0.021*	
C4	0.8362 (2)	0.5378 (3)	0.98853 (7)	0.0190 (4)	
H4	0.8862	0.4220	1.0056	0.023*	
C5	0.7328 (2)	0.6403 (3)	1.02288 (7)	0.0172 (4)	
C6	0.6650 (2)	0.8189 (3)	0.99909 (8)	0.0200 (4)	
H6	0.6001	0.8901	1.0235	0.024*	
C7	0.69427 (19)	0.8905 (3)	0.93914 (7)	0.0172 (4)	
H7	0.6489	1.0099	0.9233	0.021*	
C8	0.7330 (2)	0.3780 (3)	1.10016 (8)	0.0238 (4)	
H8A	0.6763	0.3397	1.1352	0.036*	
H8B	0.8493	0.3718	1.1154	0.036*	
H8C	0.7038	0.2906	1.0633	0.036*	
C9	0.92278 (19)	0.5941 (2)	0.63394 (7)	0.0125 (3)	
C10	1.05374 (18)	0.4757 (2)	0.60954 (7)	0.0122 (4)	
C11	1.0197 (2)	0.2936 (3)	0.57978 (7)	0.0160 (3)	
H11	0.9117	0.2493	0.5716	0.019*	
C12	1.14209 (19)	0.1759 (3)	0.56200 (7)	0.0160 (3)	
H12	1.1168	0.0551	0.5416	0.019*	
C13	1.30388 (19)	0.2427 (3)	0.57528 (7)	0.0135 (3)	
C14	1.33919 (17)	0.4281 (3)	0.60267 (6)	0.0160 (3)	
H14	1.4464	0.4749	0.6093	0.019*	
C15	1.2158 (2)	0.5421 (3)	0.61986 (7)	0.0148 (3)	
H15	1.2406	0.6651	0.6386	0.018*	
C16	1.4086 (2)	−0.0503 (3)	0.53119 (7)	0.0191 (4)	
H16A	1.5118	−0.1095	0.5271	0.029*	
H16B	1.3448	−0.0312	0.4880	0.029*	
H16C	1.3501	−0.1347	0.5566	0.029*	
C17	1.20276 (19)	1.0299 (3)	0.74076 (7)	0.0156 (3)	
H17	1.2125	0.9018	0.7261	0.019*	
C18	1.34326 (19)	1.1433 (3)	0.75550 (7)	0.0143 (3)	
H18	1.4452	1.0910	0.7518	0.017*	
C19	1.32986 (18)	1.3358 (2)	0.77577 (7)	0.0119 (3)	
C20	1.17472 (17)	1.4047 (3)	0.78314 (6)	0.0132 (3)	
H20	1.1614	1.5323	0.7975	0.016*	
C21	1.04258 (19)	1.2809 (3)	0.76878 (7)	0.0145 (3)	
H21	0.9402	1.3273	0.7746	0.017*	
C22	1.47067 (19)	1.4762 (2)	0.78517 (7)	0.0135 (3)	

### Atomic displacement parameters (Å^2^)

**Table:**

	*U*^11^	*U*^22^	*U*^33^	*U*^12^	*U*^13^	*U*^23^
Co1	0.00979 (9)	0.00942 (10)	0.01397 (8)	−0.00058 (10)	0.00201 (6)	−0.00009 (9)
O1	0.0127 (5)	0.0116 (6)	0.0158 (5)	0.0014 (5)	0.0036 (4)	−0.0003 (4)
O2	0.0120 (6)	0.0131 (6)	0.0190 (5)	0.0013 (5)	0.0042 (4)	0.0016 (5)
O3	0.0236 (7)	0.0243 (8)	0.0170 (5)	0.0001 (6)	0.0068 (5)	0.0039 (5)
O4	0.0115 (5)	0.0136 (6)	0.0223 (5)	−0.0021 (5)	0.0046 (4)	−0.0018 (5)
O5	0.0133 (5)	0.0123 (6)	0.0191 (5)	−0.0025 (5)	0.0051 (4)	−0.0027 (5)
O6	0.0133 (5)	0.0146 (7)	0.0201 (5)	0.0021 (5)	0.0033 (4)	−0.0031 (5)
O7	0.0144 (6)	0.0106 (6)	0.0237 (5)	0.0000 (5)	0.0057 (4)	−0.0008 (5)
O8	0.0124 (6)	0.0125 (7)	0.0192 (5)	−0.0017 (5)	0.0029 (4)	−0.0006 (5)
O9	0.0139 (6)	0.0125 (7)	0.0246 (6)	−0.0031 (5)	−0.0029 (5)	0.0033 (5)
N1	0.0124 (6)	0.0120 (7)	0.0137 (5)	−0.0005 (6)	0.0024 (5)	0.0007 (5)
N2	0.0111 (6)	0.0087 (9)	0.0324 (7)	−0.0007 (6)	0.0029 (5)	−0.0019 (7)
C1	0.0066 (7)	0.0138 (8)	0.0178 (7)	−0.0038 (6)	0.0000 (6)	−0.0006 (6)
C2	0.0121 (7)	0.0141 (9)	0.0153 (6)	−0.0031 (7)	0.0010 (5)	0.0003 (6)
C3	0.0148 (8)	0.0194 (10)	0.0174 (7)	0.0026 (7)	0.0031 (6)	−0.0007 (7)
C4	0.0178 (8)	0.0183 (10)	0.0202 (7)	0.0026 (7)	0.0015 (6)	0.0033 (7)
C5	0.0148 (8)	0.0226 (10)	0.0139 (6)	−0.0037 (7)	0.0019 (6)	0.0004 (6)
C6	0.0211 (9)	0.0204 (10)	0.0201 (7)	0.0019 (7)	0.0076 (6)	−0.0029 (7)
C7	0.0174 (7)	0.0140 (11)	0.0206 (7)	−0.0004 (7)	0.0042 (6)	−0.0006 (6)
C8	0.0236 (9)	0.0289 (13)	0.0188 (7)	−0.0002 (8)	0.0030 (6)	0.0080 (7)
C9	0.0140 (8)	0.0123 (9)	0.0112 (6)	0.0002 (6)	0.0017 (6)	0.0016 (6)
C10	0.0117 (7)	0.0137 (10)	0.0115 (6)	0.0007 (6)	0.0025 (5)	0.0023 (5)
C11	0.0116 (8)	0.0175 (9)	0.0189 (7)	−0.0026 (7)	0.0025 (6)	−0.0010 (7)
C12	0.0167 (8)	0.0138 (9)	0.0174 (7)	−0.0025 (7)	0.0029 (6)	−0.0037 (6)
C13	0.0150 (8)	0.0143 (9)	0.0116 (6)	0.0008 (7)	0.0031 (5)	0.0004 (6)
C14	0.0126 (6)	0.0170 (8)	0.0187 (6)	−0.0038 (9)	0.0034 (5)	−0.0018 (8)
C15	0.0175 (8)	0.0120 (9)	0.0151 (6)	−0.0023 (7)	0.0031 (6)	−0.0033 (6)
C16	0.0222 (8)	0.0158 (10)	0.0191 (6)	0.0021 (8)	0.0035 (6)	−0.0044 (7)
C17	0.0151 (8)	0.0119 (9)	0.0198 (7)	0.0013 (7)	0.0030 (6)	−0.0024 (6)
C18	0.0101 (7)	0.0135 (9)	0.0200 (7)	0.0021 (7)	0.0047 (6)	−0.0011 (6)
C19	0.0121 (7)	0.0126 (8)	0.0116 (6)	−0.0016 (6)	0.0031 (5)	0.0008 (6)
C20	0.0139 (7)	0.0089 (10)	0.0175 (6)	0.0032 (7)	0.0048 (5)	−0.0019 (6)
C21	0.0122 (8)	0.0142 (9)	0.0178 (7)	0.0008 (7)	0.0047 (6)	−0.0010 (6)
C22	0.0136 (7)	0.0136 (10)	0.0145 (6)	−0.0018 (6)	0.0052 (5)	−0.0022 (5)

### Geometric parameters (Å, °)

**Table:**

Co1—O1	2.1338 (11)	C6—C5	1.392 (3)
Co1—O2	2.2301 (11)	C6—H6	0.9300
Co1—O5	2.0506 (11)	C7—C6	1.383 (2)
Co1—O8	2.1394 (12)	C7—H7	0.9300
Co1—O9	2.0317 (13)	C8—H8A	0.9600
Co1—N1	2.1077 (13)	C8—H8B	0.9600
O1—C1	1.276 (2)	C8—H8C	0.9600
O2—C1	1.260 (2)	C10—C9	1.502 (2)
O3—C5	1.3662 (19)	C10—C11	1.389 (2)
O3—C8	1.431 (2)	C10—C15	1.395 (2)
O4—C9	1.2729 (19)	C11—H11	0.9300
O5—C9	1.258 (2)	C12—C11	1.387 (2)
O6—C13	1.3730 (19)	C12—C13	1.395 (2)
O6—C16	1.437 (2)	C12—H12	0.9300
O7—C22	1.237 (2)	C14—C13	1.392 (3)
O8—H81	0.83 (2)	C14—C15	1.375 (2)
O8—H82	0.89 (3)	C14—H14	0.9300
O9—H91	0.79 (2)	C15—H15	0.9300
O9—H92	0.91 (2)	C16—H16A	0.9600
N1—C17	1.344 (2)	C16—H16B	0.9600
N1—C21	1.342 (2)	C16—H16C	0.9600
N2—C22	1.333 (2)	C17—C18	1.385 (2)
N2—H2A	0.79 (2)	C17—H17	0.9300
N2—H2B	0.91 (2)	C18—H18	0.9300
C2—C1	1.489 (2)	C19—C18	1.385 (2)
C2—C3	1.387 (2)	C19—C20	1.398 (2)
C2—C7	1.398 (2)	C20—H20	0.9300
C3—C4	1.392 (2)	C21—C20	1.371 (2)
C3—H3	0.9300	C21—H21	0.9300
C4—H4	0.9300	C22—C19	1.493 (2)
C5—C4	1.386 (2)		
			
O1—Co1—O2	60.32 (4)	C6—C7—H7	119.8
O1—Co1—O8	88.94 (4)	O3—C8—H8A	109.5
O5—Co1—O1	96.83 (4)	O3—C8—H8B	109.5
O5—Co1—O2	156.69 (4)	O3—C8—H8C	109.5
O5—Co1—O8	92.47 (5)	H8A—C8—H8B	109.5
O5—Co1—N1	90.44 (5)	H8A—C8—H8C	109.5
O8—Co1—O2	91.64 (5)	H8B—C8—H8C	109.5
O9—Co1—O1	153.01 (5)	O4—C9—C10	117.72 (14)
O9—Co1—O2	95.22 (5)	O5—C9—O4	124.05 (14)
O9—Co1—O5	108.09 (5)	O5—C9—C10	118.23 (13)
O9—Co1—O8	79.99 (5)	C11—C10—C9	121.43 (14)
O9—Co1—N1	92.80 (5)	C11—C10—C15	118.24 (15)
N1—Co1—O1	97.30 (5)	C15—C10—C9	120.23 (14)
N1—Co1—O2	88.29 (5)	C10—C11—H11	119.1
N1—Co1—O8	172.76 (5)	C12—C11—C10	121.90 (15)
C1—O1—Co1	91.92 (10)	C12—C11—H11	119.1
C1—O2—Co1	87.97 (10)	C11—C12—C13	118.61 (16)
C5—O3—C8	117.25 (14)	C11—C12—H12	120.7
C9—O5—Co1	127.62 (10)	C13—C12—H12	120.7
C13—O6—C16	118.14 (12)	O6—C13—C14	115.31 (13)
Co1—O8—H81	93.8 (15)	O6—C13—C12	124.54 (15)
Co1—O8—H82	109.5 (15)	C14—C13—C12	120.15 (15)
H81—O8—H82	108 (2)	C13—C14—H14	119.9
Co1—O9—H91	117.3 (17)	C15—C14—C13	120.10 (14)
Co1—O9—H92	134.2 (12)	C15—C14—H14	119.9
H91—O9—H92	108 (2)	C10—C15—H15	119.6
C17—N1—Co1	121.84 (11)	C14—C15—C10	120.90 (16)
C21—N1—Co1	120.64 (11)	C14—C15—H15	119.6
C21—N1—C17	117.40 (14)	O6—C16—H16A	109.5
C22—N2—H2A	125.4 (14)	O6—C16—H16B	109.5
C22—N2—H2B	118.0 (14)	O6—C16—H16C	109.5
H2A—N2—H2B	117 (2)	H16A—C16—H16B	109.5
O1—C1—C2	118.41 (15)	H16A—C16—H16C	109.5
O2—C1—O1	119.79 (14)	H16B—C16—H16C	109.5
O2—C1—C2	121.77 (15)	N1—C17—C18	122.89 (16)
C3—C2—C7	118.77 (14)	N1—C17—H17	118.6
C3—C2—C1	120.01 (15)	C18—C17—H17	118.6
C7—C2—C1	121.09 (15)	C17—C18—H18	120.5
C2—C3—C4	121.42 (16)	C19—C18—C17	119.09 (15)
C2—C3—H3	119.3	C19—C18—H18	120.5
C4—C3—H3	119.3	C18—C19—C20	118.15 (15)
C3—C4—H4	120.6	C18—C19—C22	122.94 (15)
C5—C4—C3	118.90 (17)	C20—C19—C22	118.75 (15)
C5—C4—H4	120.6	C19—C20—H20	120.6
O3—C5—C4	123.71 (17)	C21—C20—C19	118.87 (17)
O3—C5—C6	115.79 (15)	C21—C20—H20	120.6
C4—C5—C6	120.49 (15)	N1—C21—C20	123.53 (15)
C7—C6—C5	119.92 (16)	N1—C21—H21	118.2
C7—C6—H6	120.0	C20—C21—H21	118.2
C5—C6—H6	120.0	O7—C22—N2	122.40 (15)
C2—C7—H7	119.8	O7—C22—C19	119.83 (14)
C6—C7—C2	120.36 (16)	N2—C22—C19	117.72 (15)
			
O2—Co1—O1—C1	0.09 (8)	C7—C2—C1—O2	10.6 (2)
O5—Co1—O1—C1	175.24 (9)	C3—C2—C1—O1	8.3 (2)
O8—Co1—O1—C1	−92.40 (9)	C7—C2—C1—O1	−167.51 (14)
O9—Co1—O1—C1	−27.20 (15)	C1—C2—C3—C4	−173.46 (15)
N1—Co1—O1—C1	83.91 (9)	C7—C2—C3—C4	2.4 (2)
O1—Co1—O2—C1	−0.10 (8)	C1—C2—C7—C6	173.09 (15)
O5—Co1—O2—C1	−12.36 (15)	C3—C2—C7—C6	−2.8 (2)
O8—Co1—O2—C1	87.75 (9)	C2—C3—C4—C5	0.7 (3)
O9—Co1—O2—C1	167.84 (9)	O3—C5—C4—C3	175.05 (15)
N1—Co1—O2—C1	−99.50 (9)	C6—C5—C4—C3	−3.5 (2)
O1—Co1—O5—C9	60.93 (12)	C7—C6—C5—O3	−175.47 (15)
O2—Co1—O5—C9	71.64 (17)	C7—C6—C5—C4	3.2 (2)
O8—Co1—O5—C9	−28.29 (12)	C2—C7—C6—C5	0.0 (2)
O9—Co1—O5—C9	−108.57 (12)	C11—C10—C9—O4	5.9 (2)
N1—Co1—O5—C9	158.34 (12)	C11—C10—C9—O5	−174.93 (13)
O1—Co1—N1—C17	75.29 (12)	C15—C10—C9—O4	−170.39 (13)
O1—Co1—N1—C21	−108.72 (11)	C15—C10—C9—O5	8.8 (2)
O2—Co1—N1—C17	135.07 (12)	C9—C10—C11—C12	−174.61 (14)
O2—Co1—N1—C21	−48.93 (11)	C15—C10—C11—C12	1.7 (2)
O5—Co1—N1—C17	−21.65 (12)	C9—C10—C15—C14	174.66 (14)
O5—Co1—N1—C21	154.35 (11)	C11—C10—C15—C14	−1.7 (2)
O9—Co1—N1—C17	−129.79 (12)	C13—C12—C11—C10	0.8 (2)
O9—Co1—N1—C21	46.21 (12)	C11—C12—C13—O6	175.92 (14)
Co1—O1—C1—O2	−0.17 (14)	C11—C12—C13—C14	−3.3 (2)
Co1—O1—C1—C2	177.95 (12)	C15—C14—C13—O6	−175.95 (13)
Co1—O2—C1—O1	0.16 (14)	C15—C14—C13—C12	3.3 (2)
Co1—O2—C1—C2	−177.89 (13)	C13—C14—C15—C10	−0.8 (2)
C8—O3—C5—C4	−8.5 (2)	N1—C17—C18—C19	−1.6 (2)
C8—O3—C5—C6	170.10 (14)	C20—C19—C18—C17	2.6 (2)
Co1—O5—C9—O4	19.3 (2)	C22—C19—C18—C17	−172.69 (13)
Co1—O5—C9—C10	−159.84 (9)	C18—C19—C20—C21	−1.3 (2)
C16—O6—C13—C12	5.3 (2)	C22—C19—C20—C21	174.22 (13)
C16—O6—C13—C14	−175.44 (13)	N1—C21—C20—C19	−1.2 (2)
Co1—N1—C17—C18	175.31 (11)	O7—C22—C19—C18	151.80 (15)
C21—N1—C17—C18	−0.8 (2)	N2—C22—C19—C18	−25.7 (2)
Co1—N1—C21—C20	−173.91 (11)	O7—C22—C19—C20	−23.5 (2)
C17—N1—C21—C20	2.3 (2)	N2—C22—C19—C20	158.99 (13)
C3—C2—C1—O2	−173.63 (14)		

### Hydrogen-bond geometry (Å, °)

**Table:**

*D*—H···*A*	*D*—H	H···*A*	*D*···*A*	*D*—H···*A*
N2—H2A···O2^i^	0.79 (3)	2.11 (3)	2.877 (2)	164.0 (17)
N2—H2B···O1^ii^	0.91 (3)	2.16 (3)	3.050 (2)	167 (2)
O8—H81···O4	0.83 (3)	1.84 (3)	2.6577 (17)	167 (3)
O8—H82···O7^iii^	0.89 (2)	1.86 (3)	2.7427 (16)	172 (2)
O9—H91···O6^iv^	0.786 (19)	2.078 (19)	2.8384 (16)	163 (2)
O9—H92···O4^v^	0.91 (3)	1.72 (3)	2.6307 (18)	174.1 (15)
C8—H8A···O7^vi^	0.96	2.53	3.466 (2)	166
C16—H16B···O4^vii^	0.96	2.52	3.4752 (18)	171

Symmetry codes: (i) *x*+1, *y*, *z*; (ii) *x*+1, *y*+1, *z*; (iii) *x*−1, *y*−1, *z*; (iv) *x*−1, *y*+1, *z*; (v) *x*, *y*+1, *z*; (vi) −*x*+2, *y*−3/2, −*z*+2; (vii) −*x*+2, *y*−1/2, −*z*+1.
